# Foods, the Best Way to Take Antioxidant Natural Products

**DOI:** 10.3390/foods10010019

**Published:** 2020-12-23

**Authors:** Maria Eduarda Machado Araújo, Alice Martins

**Affiliations:** 1 CQE and Department of Chemistry and Biochemistry, Faculty of Sciences, University of Lisbon, Campo Grande, 1749-016 Lisboa, Portugal; 2 CQE-Faculty of Sciences, University of Lisbon, Campo Grande, 1749-016 Lisboa, Portugal; aimartins@fc.ul.pt

Antioxidants are powerful compounds that help the body to destroy the excess of endogenous radical species responsible for many severe conditions like neurodegenerative, inflammatory, and cardiovascular impairments, and even some forms of cancer. Antioxidants present in foods that belong to the normal diet of any population are the most natural and easy way to provide the body with antioxidants.

In this special issue, 14 high scientific quality articles present the antioxidant properties of different foods or food ingredients, from beverages to baked products and spices.

Beverages are quite appreciated as a meal accompaniment or by themselves. The antioxidant properties of three beverages are described in this issue. Klarić et al. [1] present the phenolic composition and content of blackberry wines, a traditional alcoholic beverage of Croatia, as well their antioxidant activity. In their work, the authors found that the antioxidant activity of the investigated blackberry wines was proportional to the measured concentrations of both total polyphenolics and individual groups (tannins, non-flavonoids) of these bioactive compounds. The in vivo antioxidant properties of another beverage, the Chinese tea (*Camellia sinensis*), very popular not only in China but all around the world, was investigated by Cao et al. [2]. Using a mouse model with acute alcohol-induced liver injury, the authors found that the in vivo antioxidant activity of dark tea was stronger than that of green tea, which was opposite to the results of in vitro studies. They also found that the contents of epicatechin, gallocatechin gallate, and chlorogenic acid contribute, besides to the antioxidant properties, to a hepatoprotective action. Another beverage prepared from *Camellia sinensis*, Matcha tea, which is a powdered type of Japanese green tea of the Tencha type, was investigated by Jakubczyk et al. [3]. The authors found that this beverage is characterized by particularly high contents of rutin, polyphenols and vitamin C, responsible for the high antioxidant potential. They also found that its properties are, however, influenced by factors such as the time of harvest and the temperature of brewing.

Fruits are also a good source of antioxidant compounds. Interestingly, grape seeds, a by-product of red winemaking, obtained from four Italian red grape cultivars were investigated by Bosso et al. [4]. The authors performed a preliminary characterization of the polyphenolic profile and of the antioxidant properties of these seeds after different periods of maceration, and conclude that they can be a precious source of polyphenolic compounds, which can be used at an industrial level. Zitouni et al. [5] studied the strawberry tree fruits among twelve genotypes from Morocco. Besides their antioxidant properties, the authors identified seventeen phenolic compounds responsible for this activity. Small berries are becoming very popular in the Western countries given their antioxidant and anti-inflammatory activity. Zorzi et al. [6] investigated these properties in eleven different berry fruits cultivated in the North West of Italy. The authors found that blueberries and blackberries have the highest concentration in polyphenols, while the berry with the lowest polyphenol abundance was the white gooseberry, and suggest the use of the former as the best natural antioxidant for food and health purposes among the studied berries. Hawthorn berry (*Crataegus* spp.) can be considered a rich source of antioxidants, due to their high phenolic composition and some well-known antioxidant compounds. Alirezalu et al. [7] studied 15 fruit specimens collected from wild-growing *Crataegus* species from seven provinces of Iran and evaluated their antioxidant activity and described the composition of phenolic compounds in all the 15 analysed species.

Buckwheat is a plant cultivated for its fruit seeds that are similar to cereals. Buckwheat flour extracts were studied by Jin et al. [8], which found that hydrothermal treatment enhances antioxidant activity and intestinal absorption of rutin, the main compound responsible for this activity. Cereal sprouts have been paid attention due to the reports that they have increased nutritional values but there are few studies on the use of sprouted wheat flours for the production of flat, an unleavened bread-like product. Alfeo et al. [9] studied the effects of baking time and temperature on phenolic compounds content of fresh sprouts added to bread-like products and found that, although the level of the total polyphenols remained substantially unchanged during the process, the phenolic acids content and profile were affected by baking conditions.

Sweet potato (*Ipomoea batatas* L.) is a food crop widely consumed around the world. Usually, only its tuber is eaten, however the young leaves and petiole (shoot) can be consumed as greens. Zhang et al. [10] studied the antioxidant activity of sweat potato leaves and reported its chemical characterization.

Flowers are usually more appreciated for their aesthetic value. Nevertheless, edible flowers can be used in culinary to produce exquisite and delicate dishes, sweets or drinks. Xiang et al. [11] investigated the entire phenolic profiles and antioxidant activities of different organs of the edible tree peony flowers (Fengdan Bai) from China. These authors found that flowers exhibited high antioxidant activities that can be attributed to their phenolic compounds. Furthermore, they found that a phenolic extract of these flowers increased cell viability in an oxidative damage model of Caco-2 cells, thus having therapeutic effects on the studied intestinal model.

Thai *Curcuma* species are widely consumed in Thailand besides turmeric (*Curcuma longa* a well-known spice. Burapan et al. [12] studied twenty-three species of Thai *Curcuma*, and have isolated and identified the main secondary metabolites and evaluated the antioxidant activity of ethanolic extracts.

Last, but not least, two studies on foods from marine origin are also included in this special issue. Pinheiro et al. [13] showed that a gourmet pâté made from limpets could be preserved from oxidation and microbial contamination, maintaining the quality parameters like colour, texture and pH, using a water extract of strawberry tree fruits as a natural antioxidant. Francisco et al. [14] evaluated the total phenolic content and the antioxidant activity of the brown seaweed *Fucus spiralis* and studied its bioaccessibility in an in vitro model.

Finally, it can be said that edible parts of many plants, from roots to flowers, and marine organisms that are consumed as foods, can provide natural antioxidants that helps the body to fight oxidative damage contributing to the maintenance of human health.
